# ISS alone, is not sufficient to correctly assign patients post hoc to trauma team requirement

**DOI:** 10.1007/s00068-020-01410-4

**Published:** 2020-06-16

**Authors:** Christian Waydhas, Dan Bieler, Uwe Hamsen, Markus Baacke, Rolf Lefering, The TraumaRegister DGU

**Affiliations:** 1 Ruhr-Universität-Bochum, Universitätsstrasse 150, 44801 Bochum, Germany; 2 BG Universitätsklinikum Bergmannsheil Bochum, Klinik und Poliklinik für Chirurgie, Bürkle-de-la-Camp-Platz 1, 44789 Bochum, Germany; 3 Medical Faculty of the University Duisburg-Essen, University Hospital, Hufelandstr. 55, 45147 Essen, Germany; 4 Department for Trauma Surgery and Orthopedics, Reconstructive Surgery, Hand Surgery, Burn Medicine, German Armed Forces Central Hospital Koblenz, Ruebenacher Straße 170, 56072 Koblenz, Germany; 5 Department for Orthopedics and Trauma Surgery, Heinrich Heine University Hospital Duesseldorf, Moorenstr. 5, 40225 Düsseldorf, Germany; 6 Klinik für Unfall- und Wiederherstellungschirurgie, Krankenhaus der Barmherzigen Brüder, Nordallee 1, 54292 Trier, Germany; 7 Institut für Forschung in der Operativen Medizin (IFOM), Abteilung Statistik und Registerforschung, Universität Witten/Herdecke, Ostmerheimer Str.200, Haus 38, 51109 Cologne, Germany; 8 Committee on Emergency Medicine, Intensive Care and Trauma Management (Sektion NIS) of the German Trauma Society (DGU), Berlin, Germany

**Keywords:** Trauma team, Trauma team activation, Triage, Quality control, Wounds and injuries, Trauma centers, Hospital, Emergency service

## Abstract

**Purpose:**

An injury severity score (ISS) ≥ 16 alone, is commonly used post hoc to define the correct activation of a trauma team. However, abnormal vital functions and the requirement of life-saving procedures may also have a role in defining trauma team requirement post hoc. The aim of this study was to describe their prevalence and mortality in severely injured patients and to estimate their potential additional value in the definition of trauma team requirement as compared to the definition based on ISS alone.

**Methods:**

Retrospective analysis of a trauma registry including patients with trauma team activation from the years 2009 until 2015, who were 16 years of age or older and were brought to the trauma center directly from the scene. Patients were divided into a group with an ISS ≥ 16 vs. ISS < 16. For analysis a predefined list of abnormal vital functions and life-saving interventions was used.

**Results:**

58,723 patients were included in the study (*N* = 32,653 with ISS ≥ 16; *N* = 26,070 with ISS < 16). From the total number of patients that required life-saving procedures or presented with abnormal vital functions 29.1% were found in the ISS < 16 group. From the ISS < 16 group, 36.7% of patients required life-saving procedures or presented with abnormal vital signs. The mortality of those was 8.1%.

**Conclusions:**

Defining the true requirement of trauma team activation post hoc by using ISS ≥ 16 alone does miss a considerable number of subjects who require life-saving interventions or present with abnormal vital functions. Therefore, life-saving interventions and abnormal vital functions should be included in the definitions for trauma team requirement. Further studies have to evaluate, which life-saving procedures and abnormal vital functions are most relevant.

## Background

To measure the quality of trauma team activation some post hoc definition is necessary to define, whether the activation of a trauma team was correct or not. Many researchers and quality controllers use the Injury Severity Score (ISS) alone, to differentiate between patients who require a trauma team (ISS ≥ 16) or not (ISS < 16) [1–16]. ISS is fairly easy to calculate, can be automatically retrieved from electronic records and it appears self-evident that a higher injury severity correlates with worse outcome and that more severely injured patients may benefit from a specialized trauma team.

However, other definitions of correct trauma team activation have been suggested by the American College of Surgeons [17, 18] and others [19–31] such as the requirement of intensive care unit (ICU) treatment, death within a certain time after admission, emergency interventions, emergency surgeries, life-saving procedures and abnormal vital functions. Recently, a list of criteria has been proposed to define trauma team requirement based predominantly on life-saving procedures and abnormal vital signs [32]. The rationale behind these suggestions is the assumption that regardless of the injury severity, the presence of abnormal vital functions or the need for life-saving procedures are among the most important reasons for the requirement of a trauma team. These calculations are always retrospective, when the diagnoses, therapeutic interventions, course and outcome of the patient are known. They do not intend to guide the field triage or trauma team activation but to evaluate in retrospect the correctness of the field triage or trauma team alert decisions.

Due to the lack of a generally accepted definition it is not surprising that the criteria used in the literature do vary considerably [33–35] and accuracy and miss-triage calculations differ substantially, depending on the criteria used.

The aim of this study was to describe the prevalence and mortality of life-saving procedures and abnormal vital signs in severely injured patients with an ISS < 16 and ISS ≥ 16 and to estimate their potential additional value in the definition of trauma team requirement as compared to the ISS-based definition alone.

## Methods

### Study design and setting

The TraumaRegister DGU^®^ of the German Trauma Society (Deutsche Gesellschaft für Unfallchirurgie, DGU) was founded in 1993. The aim of this multi-center database is a pseudonymized and standardized documentation of severely injured patients.

Data are collected prospectively in four consecutive time phases from the site of the accident until discharge from hospital: (A) pre-hospital phase, (B) emergency room and initial surgery, (C) intensive care unit and (D) discharge. The documentation includes detailed information on demographics, injury pattern, comorbidities, pre- and in-hospital management, course on intensive care unit, relevant laboratory findings including data on transfusion and outcome of each individual. The inclusion criterion is trauma-related admission to hospital via emergency room with subsequent intensive care unit or intermediate care unit (ICU/IMC) care or reaching the hospital with vital signs and dying before admission to the intensive care unit.

The infrastructure for documentation, data management, and data analysis is provided by AUC—Academy for Trauma Surgery (AUC—Akademie der Unfallchirurgie GmbH), a company affiliated to the German Trauma Society. The scientific leadership is provided by the Committee on Emergency Medicine, Intensive Care and Trauma Management (Sektion NIS) of the German Trauma Society. The participating hospitals submit their pseudonymized data into a central database via a web-based application. Scientific data analysis is approved according to a peer review procedure and laid down in the publication guideline of TraumaRegister DGU^®^ [36]. The participating hospitals are primarily located in Germany (90%), but a rising number of hospitals of other countries contribute data as well (at the moment from Austria, Belgium, China, Finland, Luxembourg, Slovenia, Switzerland, The Netherlands, and the United Arab Emirates). Currently, about 33,000 cases from more than 650 hospitals are entered into the database per year.

Participation in TraumaRegister DGU^®^ is voluntary. For hospitals associated with TraumaNetzwerk DGU^®^, however, the entry of at least a basic data set is obligatory for reasons of quality assurance. In addition to the basic data set, the participating trauma centers may enter data into the more comprehensive standard data set. It is at the discretion of the participating trauma centers which type of dataset they use. However, all of their patients will then be documented in the one dataset chosen.

The present study is in line with the publication guidelines of the TraumaRegister DGU^®^ [36] and registered as TR-DGU project ID 2016-013.

### Selection of patients

Trauma emergency room treatment and trauma team activation is triggered by the prehospital emergency service using established field triage criteria [17, 37]. If a patient did not meet field triage criteria but was considered to require a trauma team by the hospital admission triage system or at the discretion of the receiving emergency physician, the trauma team was also activated. According to the regulations for trauma centers in Germany [38] treatment in the trauma emergency room comprises the activation of the trauma team.

**Fig. 1:**
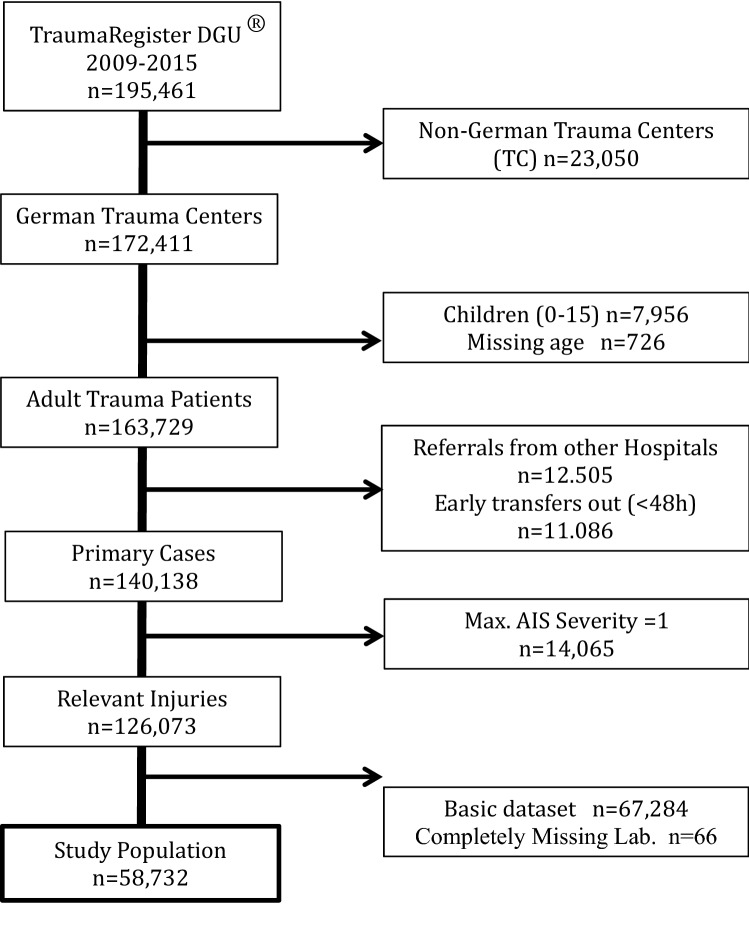
Patient selection

The basis of our analysis were the patients from the years 2009 until 2015. We included only patients from Germany who were 16 years of age or older and were brought to any of the participating trauma centers directly from the scene. We excluded patients who were transferred to another hospital within the first 48 h because of missing outcome data. We also excluded transfer-in cases because patients were pretreated and initial physiology was missing. Cases with marginal injuries (maximum AIS of 1) were also excluded. Finally, cases documented with the basic dataset had to be excluded since not all emergency interventions and laboratory values (see below) are available in the basic data set. Finally, a further 66 cases were excluded due to completely missing laboratory values. The final patient group consisted of 58,723 cases (Fig. 1).

### Measurements

The patients were divided into a group with a high (ISS ≥ 16) and a low (ISS < 16) injury severity.

To identify other indicators for the requirement of trauma team activation we reviewed additional indicators of major trauma as suggested by the American College of Surgeons Committee on Trauma [39] and performed a Medline research as previously published [32] using the terms “trauma team activation” and identified additional secondary publications from the literature screened. The search resulted in six categories of life-saving procedures and nine criteria using abnormal vital signs that could be extracted from the TraumaRegister DGU^®^ database. The following life-saving procedures (with definitions in parenthesis) were used: advanced airway (endotracheal intubation, supraglottic airway, surgical airway), catecholamines (epinephrine, norepinephrine, dobutamine administration), chest tube (any type of pleural decompression), emergency operation or intervention in the emergency department or before transfer to the ICU (operative cerebrospinal fluid drainage, cranial decompression, laminectomy, thoracotomy, laparotomy, revascularization, embolization, external pelvic or extremity stabilization with external fixator), transfusion (any administration of packed red blood cells in the emergency room) and CPR (cardiovascular resuscitation with chest compressions). The abnormal vital signs that were used comprise Glasgow Coma Scale (GCS) ≤ 8, GCS 9–13, shock index (heart rate/systolic blood pressure) > 0.9, base excess ≤ − 6 mmol/l, systolic blood pressure ≤ 90 mmHg, systolic blood pressure < 110 mmHg in elderly patients aged > 65 year, hemoglobin < 10 g/dl, pulse oximetry (SpO_2_) < 90%.

### Analysis

Variables were analyzed using descriptive statistics (percentages and frequencies) and central tendency measures (mean with standard deviation and median). SPSS Version 22 for Windows^®^ (IBM Inc., Armonk, NY) was used for the statistical analysis. The study was approved by the Ethics Committee of the Ruhr University Bochum (reference number 19-6589-BR).

## Results

Overall, 58,723 patients are the basis of our study. The characteristics are shown in Table 1. Classification of patients by ISS yielded 32,653 patients with an ISS ≥ 16 and 26,070 patients with an ISS below 16.

**Table 1:** Characteristics of the study group

Age, years	49.7/49/SD 20.8
Gender (M/F), %	71.1/28.9
ISS, points	17/9–25
Blunt trauma, %	95.5
Mortality, %	11.7
Mechanism of injury, %	
Motor vehicle accident	23.2
Motorcycle accident	13.6
Bicycle accident	8.6
Pedestrian	6.7
Fall > 3 m	17.2
Fall ≤ 3 m	18.4
Blunt hit	2.9
Gunshot	0.6
Stabbing	1.7
Others	5.0
Level of trauma center [38], %	
Level I (supra-regional)	81.4
Level II (regional)	15.9
Level III (local)	2.7

Data are given as mean/median/standard deviation (age), median and interquartile range (ISS) or percent of the whole group of 58,723 patients

Overall hospital mortality in the ISS ≥ 16 group was 19.1%. The hospital mortality with an ISS < 16 was 2.4%. Of all deaths, 90.7% occurred in the ISS ≥ 16 group and 9.3% in the ISS < 16 group.

The prevalence of the different life-saving procedures within the ISS ≥ 16 group was highest for advanced airway and lowest for CPR, while the prevalence of the different life-saving procedures in the ISS < 16 group varied between 0.5 and 20.3% (Table 2). The percentage from the total of all the respective life-saving procedures that were done in the ISS < 16 group varied between 7.3% (cardio-pulmonary resuscitation, CPR) and 21.6% (advanced airway) (Table 2). Mortality of patients in the ISS < 16 group was higher than 5%, if catecholamines, transfusion, advanced airways, emergency operations/interventions, or CPR were required.

**Table 2:** Life-saving procedures as identified as potential criteria of trauma team activation within the ISS ≥ 16 and ISS < 16 groups (in % of respective groups)

Criterion	Prevalence of criterion within ISS ≥ 16 group	Prevalence of criterion within ISS < 16 group	Percentage of all patients with the criterion in ISS ≥ 16/ISS < 16 group	Mortality within ISS ≥ 16 group	Mortality within ISS < 16 group
Advanced airway	58.5	20.3	78.4/21.6	28.8	6.8
Catecholamines	26.0	4.8	87.2/12.8	38.6	15.1
Chest tube	16.8	3.5	85.7/14.3	25.5	3.8
Emergency operation/intervention	26.0	4.8	87.3/12.7	38.6	15.1
Transfusion	17.2	2.6	89.3/10.7	31.9	9.4
CPR	4.6	0.5	92.7/7.3	87.2	67.0

While the prevalence of the respective abnormal vital functions in the ISS ≥ 16 group was below 20% (except for GCS < 8, GCS 9–13, coagulopathy, shock index), the mortality rose to more than 40% for patients who presented with almost any of the abnormal vital signs (above 30% for 7 of 9 vital signs, above 40% for 5 of 9 vital signs) (Table 3).

**Table 3:** Abnormal vital functions as identified as potential criteria of trauma team activation within the ISS ≥ 16 and ISS < 16 groups (in % of respective groups)

Criterion	Prevalence of criterion within ISS ≥ 16 group	Prevalence of criterion within ISS < 16 group	Percentage of all patients with the criterion in ISS ≥ 16/ISS < 16 group	Mortality within ISS ≥ 16 group	Mortality within ISS < 16 group
Coagulopathy	32.1	13.5	74.9/25.1	34.0	8.5
GCS ≤ 8	31.0	7.4	84.3/15.7	44.0	13.6
GCS 9–13	19.3	13.3	65.2/34.8	14.9	3.4
Shock index (heart rate/systolic blood pressure) > 0.9	21.3	7.1	78.7/21.3	29.2	6.2
Base excess ≤ − 6 mmol/l	18.6	5.9	81.8/18.3	41.9	11.3
Systolic blood pressure ≤ 90 mmHg	14.6	3.2	85.1/14.9	41.3	13.3
Hemoglobin < 10 g/dl	16.0	4.8	80.9/19.1	38.2	11.3
SpO_2_ < 90% (on admission)	7.3	2.4	78.6/21.4	44.7	14.2
Systolic blood pressure < 110 mmHg and age > 65 years	6.5	2.0	80.0/20.0	51.4	21.2

The prevalence of abnormal vital functions in the ISS < 16 group (Table 3) varied between 2.0 and 13.5%. Of all patients in the total study population who presented with a respective abnormal vital sign, a range of 14.9% (systolic blood pressure ≤ 90 mmHg) to 34.8% (GCS 9–13) occurred in the ISS < 16 group. Mortality of patients in the ISS < 16 group was more than 10% for patients presenting with a GCS ≤ 8, base excess ≤ − 6 mmol/l, systolic blood pressure ≤ 90 mmHg, hemoglobin < 10 g/dl, SpO_2_ < 90% or systolic blood pressure < 110 mmHg and age > 65 years.

**Fig. 2:**
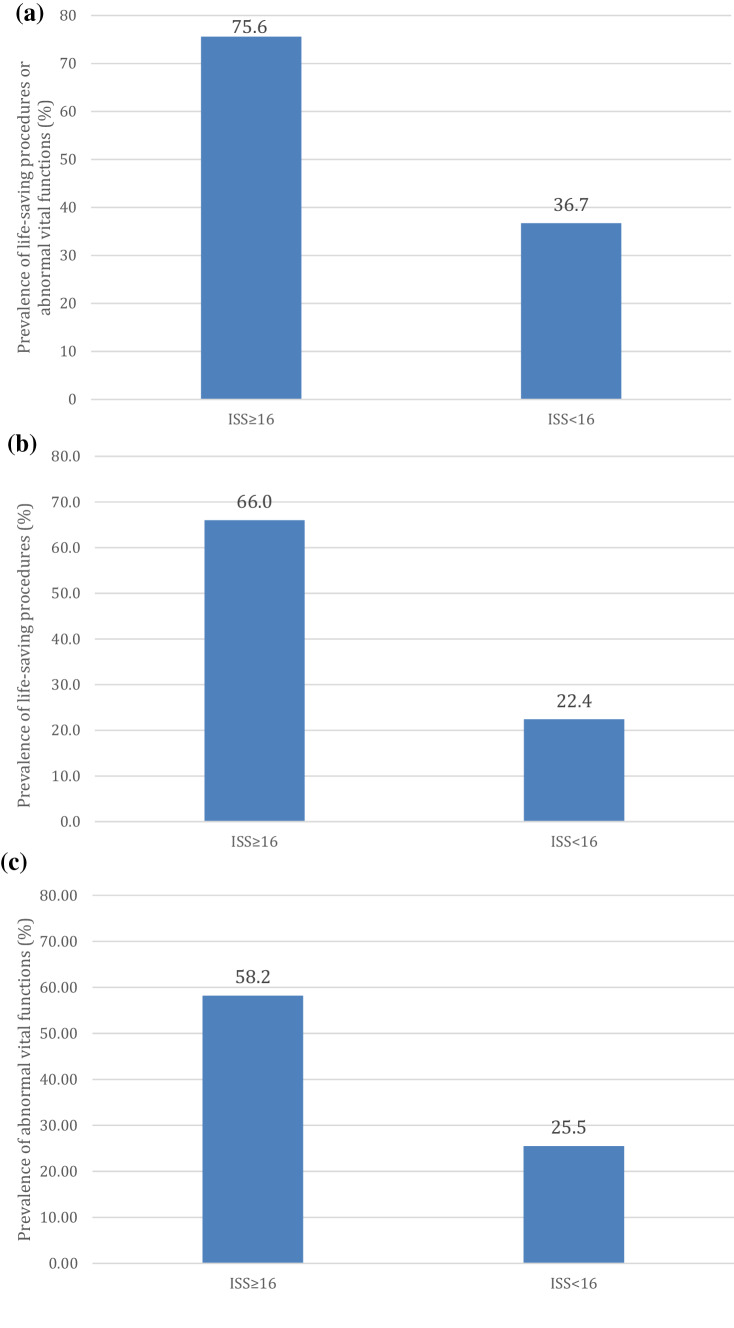
**a** Prevalence of at least one life-saving procedure or at least one abnormal vital function present within the more severely injured patients (ISS ≥ 16) and the less severely injured patients (ISS < 16). **b** Prevalence of at least one life-saving procedure within the more severely injured patients (ISS ≥ 16) and the less severely injured patients (ISS < 16). **c** Prevalence of at least one abnormal vital function within the more severely injured patients (ISS ≥ 16) and the less severely injured patients (ISS < 16)

Figure 2a shows the prevalence of at least one life-saving procedure performed, or at least one abnormal vital function present, in the ISS ≥ 16 and in the ISS < 16 groups respectively.

In the ISS ≥ 16 group the portion of patients who did neither require a life-saving procedure, nor presented with an abnormal vital function was 24.4%.

**Fig. 3:**
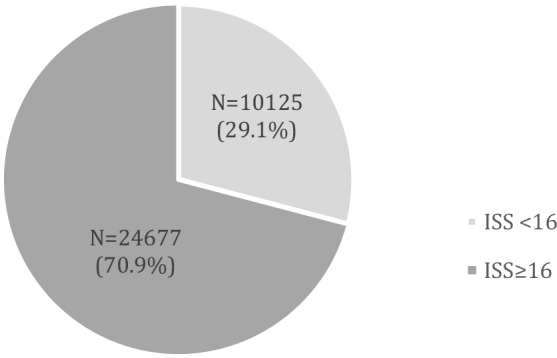
Distribution of the patients who required life-saving procedures or presented with abnormal vital signs between the groups with ISS < 16 and with ISS ≥ 16

However, 36.7% of subjects in the ISS < 16 group presented with at least one abnormal vital function or required at least one life-saving procedure (Fig. 2a). The prevalence of life-saving procedures (Fig. 2b) and of abnormal vital functions (Fig. 3c) in the respective groups was separately analyzed. Life-saving procedures were required in the ISS < 16 group in 22.4% (Fig. 2b), while the presentation with abnormal vital functions occurred in as many as 25.5% of patients (Fig. 2c).

Overall, in the whole study group there were 34,802 patients who required life-saving procedures or presented with abnormal vital signs. 10,125 (29.1%) of them were within the ISS < 16 group (Fig. 3). They would have been missed as true positives for trauma team activation if an ISS ≥ 16 would have been used. The mortality of patients with ISS < 16 but with life-saving procedures or abnormal vital signs was 8.1% (*N* = 525), or 1 out of 12.

## Discussion

In our analysis of over 58,000 severely injured patient with trauma team activation we could demonstrate that 29.1% of all patients who presented with abnormal vital functions, or who required life-saving procedures had an ISS of less than 16. They would have been miss-classified as not requiring a trauma team, if an ISS of 16 or above was used to define trauma team requirement. Furthermore, the mortality in the ISS < 16 group averaged at 8.1%, and may be as high as 21.2% (e.g. with systolic blood pressure < 110 mmHg in elderly patients aged > 65 years), depending on the type of the procedure or the type of the abnormal vital function. This compares well with the observation of Roden-Foreman et al. [40] who reported that 73 fatalities (13% of 561 patients) would have been missed by using an ISS ≥ 16 alone. This indicates that patients who require life-saving interventions or present with abnormal vital signs do have a considerable risk of dying, despite an injury severity of less than 16 ISS points. In pediatric trauma patients it has been shown that abnormal vital signs and prehospital interventions do have considerably higher odds ratio for emergency department interventions than an ISS ≥ 16 or a trauma score ≤ 8 [41]. Recently it has been shown that the need for trauma intervention had a better model fit and stronger associations with the outcomes than ISS and Revised Trauma Score [42].

Therefore, when using the criterion of an ISS ≥ 16 alone to calculate true requirement for trauma team activation a considerable number of patients will be missed that require life-saving procedures or present with abnormal vital functions. When the ISS ≥ 16 cut-off alone is used to calculate the rate of under-triage, which means that patients with an ISS < 16 would be correctly assigned to no trauma team requirement a falsely low number would result.

In the ISS ≥ 16 group more than 75% of patients either presented with abnormal vital functions or required life-saving interventions. This appears to contrast with several other studies where patients with an ISS ≥ 16 did require emergency surgical interventions in only 20–31%, chest tube insertion in 22% and transfusion in 10% of patients [13, 14]. However, these studies focused on the prevalence of single interventions, which were in a similar range as in our ISS ≥ 16 patients, but not at the total number of patients who required any of these procedures. Our prevalence of patients with abnormal vital signs (58.2%) compares well with the 52% observed in other studies [13].

There are several limitations of our study. When we look at the group with the lower injury severity (ISS < 16) the percentages (prevalence, mortality) depend on the selection criteria of the patients that form the basis for the calculations. There may be a considerable number of less severely injured patients that, although fulfilling a field triage criterion or having been treated by a trauma team, have not been included in the database of the TraumaRegister DGU^®^ because they do not fulfill TR-DGU inclusion criteria. (i.e. relevant injuries but not being admitted to an ICU and surviving). Including such patients would reduce the prevalence of life-saving interventions and of abnormal vital functions as well as the mortality within this group. For example, in one study patients without full trauma team activation (but positive field triage criteria for mechanism of injury) required urgent surgery in 1.6%, which is less than half of our less severely injured populations [33]. Including more of the less severely injured patients in our ISS < 16 group would in fact decrease the percentage of life-saving interventions and abnormal vital functions within the groups. However, the absolute number of patients with abnormal vital functions or urgent intervention would not decrease (or even slightly rise) irrespective of the group size. So, the fraction of patients with life-saving interventions or abnormal vital functions in the low injury groups as compared to the total number of patients with these criteria would essentially remain the same. Therefore, the conclusion will hold true that (at least) 29.1% of all patients with any of the criteria would be missed by using the ISS ≥ 16—definition alone. However, since we do not know the total number of patients treated with a trauma team, we cannot calculate a rate of under-triage.

Considering the different types of life-saving procedures and abnormal vital functions we did observe differences in the prevalence of single criteria as well as their mortality risk within the lower injury severity groups. It was suggested that the requirement of packed red cell transfusions as well as surgery and radiological interventions are among the most indicative variables of major trauma [40]. The mortality of these conditions in our ISS < 16 group was 15.1% (emergency operation/intervention) and 9.4% (transfusion). In pediatric trauma patients a considerable variation in the odds ratio for emergency room interventions has been described depending on the type of prehospital intervention or the type of abnormal vital function [41]. Despite the potential relative superiority of one criterion over the other we cannot decide which life-saving procedure or abnormal vital function does contribute more to identify trauma team requirement, because more than one criterion may be present in a single patient. Furthermore, not every single life-saving intervention may be really lifesaving in a particular patient. A chest tube may also be inserted for an isolated or not life-threatening pneumothorax and we cannot rule out that such subjects would be wrongly allocated to trauma team requirement. However, the mortality in patients with ISS < 16 requiring a chest tube was still 3.8% and chest tube insertion has been suggested by many authors as defining trauma team requirement [32, 41, 43]. To further optimize which life-saving procedures will or will not require trauma team activation the differential weight of criteria should be evaluated in further studies.

We did include only patients treated by a trauma team. It could be suspected that some severely injured patients could have bypassed trauma team activation by missed field triage criteria or hospital admission triage criteria. We cannot definitely rule out such missed patients, but their number is thought to be very low, since field triage criteria are applied very strictly by the prehospital personnel in Germany and triage systems are used at hospital admission for all patients not having a trauma team activation by advance notification from the prehospital emergency services.

From our data we cannot conclude whether for the post hoc definition of trauma team requirement, ISS should be completely replaced by the presence of vital functions or the requirement for urgent interventions as suggested by some [32, 43] or should be used in combination with these or other indicators like ICU admission and death [17–31]. Our data do show, however, that ISS alone is not appropriate to define trauma team requirement post hoc.

## Conclusions

Defining the true requirement of trauma team activation post hoc by using ISS ≥ 16 alone does miss a considerable number of subjects who require life-saving interventions or present with abnormal vital functions. Therefore, life-saving interventions and abnormal vital functions should be included in the definitions for trauma team requirement. Further studies have to evaluate, which life-saving procedures and abnormal vital functions are most relevant.
